# An Investigation into the Medicinal Effects of Veratria

**Published:** 1834-04-01

**Authors:** 


### An Investigation into the remarkable Medicinal Effects resulting from the External Application of Veratria. By *Alex. Turnbull,* M.D. Octavo, pp. 96. Longman and Co. 1834.

This new substance was discovered in 1819, by Messrs. Pelletier and Caventon, in the seeds of veratrium sabadella, and, about the same time, by Meysner, in Germany. Hitherto it has been obtained from veratrium sabadella, veratrium album, and colchicum autumnale. It is not a simple body, but is composed of three distinct and easily separable principles, named veratrine, sabadelline, and mono-hydrate of sabadelline. The proportions of these principles may, and do vary—hence some variety in the strength or properties of the medicine.

"The veratria of commerce is nearly white in colour, and in the form of a fine powder; it is without smell, but when accidentally or otherwise brought in contact with the mucous membrane of the nose, it induces violent and even dangerous sneezing; when applied to the conjunctiva, it produces great irritation, accompanied by an abundant flow of tears, which does not subside for some hours. Its taste is extremely acrid, but destitute of bitterness; and it acts very strongly upon the mucous membrane of the stomach and intestines: if introduced into the stomach, it proves highly emetic and purgative, for even in old subjects a quarter of a grain acts powerfully upon the bowels; and in some experiments the effects have been so violent as to shew that death would have followed the administration of a few grains. Occasionally it happens that during the preparation of veratria, some of the particles floating in the air are inhaled, and when such is the case its effects are generally purgative. M. Andral, jun. found that when it was applied immediately to the tissues, violent and speedy inflammation was the consequence; that when thrown in small quantity into the veins, it acted upon the large intestine; and that when introduced in a larger proportion, either into the veins or intestine itself, tetanus followed.* From these circumstances, veratria was very properly considered to be a medicine possessed of extreme activity, and it seems to have been some little time before it was thought safe to introduce it into practice; and although it has been employed by some individuals, yet such does not appear to have been the case to any great extent." 4.

From the medicinal qualities which will shortly be detailed, its internal use is not likely to be great. Externally, it is best applied in the form of ointment, made by rubbing from ten to twenty grains or more of the alcaloid with an ounce of hog's lard, of which the size of a large nut may be rubbed night and morning over the seat of complaint, until relief be obtained. Care should be taken that the ointment be not applied to any injured or abraded surface, otherwise considerable irritation of the part will be produced.

The effects resulting from the internal and external use of veratria are remarkably different.

"We have seen that when applied to any of the mucous membranes, even in the smallest quantity, it produces the most violent irritation, and that when introduced into the stomach it operates as an emetic and cathartic; but when rubbed upon the surface of the body to the extent of six or eight grains a day, for several weeks or even months together, no such consequences follow; for although the constitution has evidently, during the greater part of the time, been under the influence of the veratria, so far from acting in that manner, it has been observed to calm irritation, remove pain, and produce considerable elevation of spirits. The general health and appearance begin to improve, the appetite remains unimpaired or even becomes better, the patient experiences not the slightest degree of nausea; and the bowels instead of being acted upon in the manner in which the internal exhibition of the medicine would lead us to anticipate, are either altogether unaffected, or such a degree of constipation is induced as to render the use of purgatives necessary to keep them in their usual state.

When the veratria is applied externally in dropsical diseases, the emetic and cathartic effects which result from its internal employment in these affections are exchanged for a diuretic operation, so singular and beneficial as to bring about the removal of the effused fluid in a time much shorter than it can be ac-

* Magendie, Journal de Physiologie, tom. i.

complished in by any other known medicine; and it has done so in many cases after every other means had been previously tried without avail; but in diseases unattended by aqueous effusion, no effect whatever upon the kidneys has been observed." 6.

The frictions with this ointment do not irritate the skin, however long they may be employed. When more than a few grains of veratria are rubbed in, the patient generally experiences a degree of warmth and tingling in the part, and until this takes place, the peculiar effects of the medicine do not usually manifest themselves—a circumstance worthy of attention.

"After the ointment has been applied a sufficient length of time to put the constitution completely under its influence, the feeling of heat and tingling extends itself from the place where the friction may have been made, over the whole surface of the body, and in some instances involuntary twitchings of the muscles of the mouth and eyelids are induced; but these symptoms disappear so soon as the rubbing has been discontinued for a day or two, and no disagreeable consequences follow to the patient. The sensibility of the parts over which the application has been made, is increased to such a degree as to render them peculiarly susceptible of the presence of certain stimuli, particularly electricity or galvanism; these agents have in some instances been applied along with the veratria ointment, but have given rise to sensations so acute as to render their further employment almost insupportable, and that without the slightest perceptible alteration of the surface. It does not appear altogether necessary that the friction should be made exactly over the seat of the disease; for two cases have lately come under observation in which the individuals who rubbed on the ointment imbibed as much by the hand as proved sufficient to cure them of painful affections in distant parts of the body, under which they had been labouring for a considerable length of time before." 8.

Dr. T. next proceeds to a consideration of the principal diseases in which the veratia may be employed with advantage, premising that the cases are faithfully detailed, without exaggeration or concealment, in order that no extravagant expectations may be engendered by their perusal, as is too often the case with accounts of new remedies.

### I.—Affections of the Heart.

Dr. T. does not pretend that any permanent advantage has arisen from veratria where there was any structural disease in the heart, but only in those anomalous functional affections which distress the patient, alarm the friends, and excite suspicions of structural alterations.

"These diseases are generally characterised by difficulty of respiration, accompanied by cough, expectoration, inability of remaining long in a recumbent posture, startings from sleep, swelling and coldness of the extremities, palpitation, a rapid small irregular pulse, accompanied by anxiety and a sensation of pain, or rather of constriction in the region of the heart; occasional faintness and sense of suffocation. It has been successful even in worse cases: for one of angina pectoris (to be related immediately), appears to have received most marked and permanent advantage from a few rubbings with the ointment." 12.

If the above symptoms be those of functional disorder merely of the heart, we confess that we have studied in vain—for, in our practice, they are the phenomena of organic diseases. We are sincerely rejoiced, then, to learn, on Dr. Turnbull's authority, that in veratria we have a remedy for such symptoms—and even for worse symptoms than the above. It is true we should have been better satisfied if Dr. Turnbull had assured us that, on auscultic examination, he found the structure of the heart unchanged, in cases of the above description; but this mode of diagnosis does not enter much into his practice.

For affections such as are characterized by the foregoing symptoms, an ointment, with fifteen or twenty grains to the ounce of lard, is to be rubbed over the region of the heart for five minutes daily.

"In all the cases which have come under observation, a few such applications have generally proved all that was requisite either to effect a complete cure, or at least, to cause a cessation of the symptoms for a considerable time; the friction may then be employed at longer intervals, and should any slight accession take place, it may be removed by a repetition of the same means." 12.

This is the most cheering intelligence we have received since the commencement of our practice! During the action of the veratria, the pulse increases in strength and "becomes more regular—the extremities regain their natural warmth, and the swellings, when they do exist, rapidly disappear." The general health improves, and the distress and anxiety wear off. The sleep becomes refreshing—the cough and expectoration diminish daily—and the fluttering action of the heart is exchanged for regular and uninterrupted pulsation.

"Although this be the usual progress of the action of the veratria in these diseases, it nevertheless sometimes produces effects of a different kind, at least, during the time of its employment. It has happened that from one application the symptoms, and particularly the palpitation, have been increased to such a degree as to render it impossible to induce the patient to submit to a second; but what is not a little singular, the irritation has subsided in a day or two, and along with it, every trace of the disease itself has disappeared. During the whole period the constitution is under the influence of the medicine, the functions are but little altered, with the exception of the secretion of urine, which is, upon the whole, more actively carried on than at other times; and this latter circumstance, in all probability, depends upon the existence of watery effusion somewhere in the body, a condition which appears necessary before the veratria can produce a diuretic effect." 13.

In order not to interrupt the chain of our ideas by going to other subjects, and then returning, we shall introduce some cases here, in illustration of the effects of veratria in cardiac affections.

*Case* 1. A lady, 55 years of age, had been attacked seven years ago with thoracic inflammation, for which she was copiously bled. She never perfectly recovered, but had ever afterwards constant cough, scanty mucous expectoration, difficult and hurried respiration, purple lips, small, rapid, and irregular pulse, perceptible undulatory motion over the region of the heart, *and over a great part of the anterior surface of the chest*, altogether different from ordinary pulsation. "The ear applied to the chest, over the same region, distinguished the heart's action to be extended, indistinct, and undefined in character." Pain and numbness were complained of along the left arm. Except flatulence, there was no symptom of derangement of the digestive organs. She was put under the influence of small doses of tartrite of antimony, and antimonial frictions, with croton oil, were applied over the chest and down the arm, until a pretty free eruption was brought out. "Under this plan the patient, for the first time, experienced decided relief." Such debility, however, ensued, that the same remedies could not be continued. The veratria ointment was directed to be rubbed, twice a day, along the affected arm, and the result exceeded our author's anticipations.

"The cough and breathlessness, to a certain extent, subsided, and the pulse and action of the heart were greatly improved: the most decidedly beneficial effects, however, were produced upon the extremity; the pain and numbness had not altogether disappeared, but these symptoms were so much alleviated, as to induce the patient to state that, comparatively speaking, she had recovered the entire use of her arm. From this time the ointment was directed to be used every evening for about ten days, and then only occasionally, as it might be found necessary.

In about a fortnight from the first application of the veratria, the patient was able to leave her room, and walk up and down stairs with facility; and the general improvement of her health was such, that she ventured into the open air, but in consequence of incautious exposure, the symptoms returned two or three times, though by no means with the same severity as before; and when such an occurrence took place, one or two rubbings with the ointment afforded complete relief. She is now in comparative health; her general appearance is good, the pain and numbness of the arm have entirely disappeared, the circulation is much more regular than it has been for a great length of time, the cough and difficulty of respiration are almost gone, and she can now remain in the recumbent position, and enjoy a good night's rest; and the last time I saw her, she had walked about four miles without any inconvenience." 18.

The foregoing case is acknowledged by our author to be one of extensive organic disease; but whether the apparent amendment has resulted from the counter-irritation of the antimony—some temporary mitigation of the symptoms from the efforts of Nature—or the veratria, is more than we would venture to decide. We certainly have our doubts as to the efficacy of the new remedy in this case.

*Case* 2. Mr. B. aged 36, a banker's clerk, was seized ten years ago with palpitation, followed by pain and sense of stricture in the region of the heart, with irregularity of pulse. There was no cough; but the digestive functions were disordered, and the nervous system highly irritable. The veratria ointment was ordered to be used, as in the preceding case, night and morning. The patient returned in the course of a week, to report that he was "quite well." As each little box of ointment, containing an ounce, costs twelve shillings sterling, to which may be added a guinea for the Doctor's fee, it is not improbable that the banker's clerk began to calculate that this would be a costly method of cure, and reported himself as "quite well" somewhat prematurely.

*Case* 3. A lady, 36 years of age, had suffered for five years from palpitation of the heart, with much difficulty of breathing—succeeding a severe inflammation of the chest. The means employed by her physicians at Bristol had failed.

"Her eyes are suffused, her memory much impaired, and she has a considerable degree of nervous irritability. Her breathing is difficult, accompanied by slight cough and a sense of partial suffocation, along with pain across the region of the heart and down the left arm, and these feelings are materially increased by walking or any other exertion. Her pulse is irregular and quick; bowels costive; feet generally cold; and her sleep interrupted by the palpitation." 21.

Tartrite of antimony, blue-pill, and a croton-oil embrocation to the chest and arm, with quietude, produced great relief; but the palpitation returned as violently as ever upon the slightest exertion. Debility had also increased. The ointment was ordered; and frictions with croton-oil were used to the arm till a raw surface was produced, when the veratria was applied over the excoriated parts. In a day or two she was much better—and, in three weeks, she returned home "quite well."

It is hardly necessary to remark that, here again, the complication of the means leaves us in some doubt as to the merit of any particular remedy.

*Case* 4. A clergyman, aged 50, had been affected for seven years with severe palpitation, quick, irregular pulse, dyspnœa, cough, expectoration, and great anxiety.

"He was ordered to take small doses of tartar emetic, and to have a blister applied over the chest; and this treatment was pursued with considerable advantage for the time, but when it was remitted he soon returned to the same state as before. As this seemed a fair case upon which to make trial of the veratria, it was ordered to be rubbed on in the manner already described; and with its accustomed success. By making use of the frictions once every night he became gradually better, and at the end of a week considered himself quite well: he was advised, however, to continue the ointment for a little longer, and then to leave it off by degrees: this was accordingly done about six months ago, and he has remained ever since in perfect health, free from every vestige of his old complaints, and fully able for the discharge of the functions of his office." 23.

*Case* 5.—*Angina Pectoris.* Mr. W. aged 58 years, had been affected for 17 years with palpitation, and, for the last seven years, with dyspnœa in paroxysms, coming on whilst taking pedestrian exercise, presenting the usual symptoms of sternalgia. All the means employed had been ineffectual. When he applied to Dr. T. he had a peculiar purple blush on his face, with weak, quivering voice. A tremulous, confused, and irregular pulsation was heard in the region of the heart. The pulse at the wrist was irregular and intermittent. The left side of the chest appeared much larger than the right, and the intercostal spaces wider. The abdomen was distended, and an enlargement appeared in the hepatic region. The bowels were very torpid, the lower extremities a little swollen, and the urine scanty.

"A course of medicine was prescribed, consisting of laxatives combined with antacids, for the purpose of clearing out the bowels and removing the distension of the abdomen. These means were employed alone, for about a week, and then, with the addition of a little squill to the pills previously ordered, it was persevered in for a fortnight longer; at the end of which time he felt considerably better. The swelling in the legs had diminished, the digestion was improved, and, altogether, he was in a more favourable state of health than before." 25.

The veratrine ointment was now ordered to be rubbed in on the region of the heart—"and in the course of three days, the feeling of pain and constriction across the chest had entirely disappeared." The pain in the arm remaining, the frictions were applied there, and immediate relief was experienced. The patient still, from time to time, applies the ointment to the chest, and he is thereby enabled to pursue his usual avocations. The medicine exerted a strong diuretic effect in this case—and all diuretics that take a good effect are known to relieve cardiac complaints.

*Case* 6. A lady between 50 and 60, of sanguine temperament, and rather corpulent, had been ill nine years. During the first three years she suffered from dyspepsia, and for six years she had almost constant palpitation, with dyspnœa, and inability to go up stairs.

"She has violent pulsation over the region of the heart, along with an irregular and intermittent pulse, and complains, at times, of very severe pain across the chest, and stretching down the left arm: her lips are of a purplish colour; her eyes dull; her countenance sallow; and she labours under considerable nervous irritability, accompanied by impairment of the memory; she has a degree of fulness in the right side, under the margin of the ribs, and her feet are generally cold, and a little swollen. For these symptoms she had previously been treated by bleeding, blistering, purgatives, and, indeed, every thing possible appeared to have been done, without procuring any abatement in the disease." 28.

The veratria ointment was employed on the chest and down the arm every night. "In the course of a few days all the symptoms were nearly gone." The ointment was continued for a month, at the end of which time she could walk three miles with ease, "and returned home quite well." Six months have elapsed without any relapse.

Three more cases of cardiac disease or disorder are narrated, but the foregoing are sufficient. If no error has crept into either diagnosis or description, diseases of the heart may now be considered as playthings in the hands of practitioners. The worst of it is that, as it requires nearly a hundred weight of hellebore to yield an ounce of veratrine, the valleys of Switzerland and the mountains of Germany will not be able to supply us with veratria.

We now come to the second chapter, on the external application of veratria in neuralgic affections.

We are informed by our author, that the remedy in question has been employed in neuralgic pains situated in every part of the body—"and the cases which have hitherto been subjected to this plan of treatment have, almost without exception, yielded immediately to its effects, even after having resisted every other for a length of time."

"It is, however, in tic douloureux that the most remarkable and speedy change is effected in the state of the patient, for often during the continuance of the first friction the paroxysm is brought to a termination, and does not again return; and if this be not the case, the following interval is at least of much greater length than any that may have previously occurred, and the next accession of pain is much less severe and more easily removed." 36.

In facial neuralgia, we are informed, that when the pain is scattered over several branches of nerves, the symptoms are removed more easily, and by weaker ointment than when the tic is concentrated on one point. In the former case, 20 grains of veratria to the ounce of lard will be sufficient. The friction should be continued until the heat and tingling be so great as to produce an impression on the feelings of the patient equal to that arising from the disease itself, when it is to be discontinued for a short time, again to be renewed, if the neuralgic pain appears to predominate.

Where the pain is concentrated in the frontal nerve the case is more obdurate than almost any other—yet even such is found to yield to an ointment of sufficient strength, without other means.

"For the purpose of obtaining the full effect of the veratria as soon as possible in such instances, it has been used in the proportion of forty grains to an ounce of lard, and this may be done either from the very beginning of the treatment, or the quantity of the alcaloid may be augmented by five grains in each prescription until it attain to that amount. The former method is upon the whole to be preferred, because by it an immediate check is put upon the paroxysm in severe cases, without the necessity of continuing for a length of time the employment of weaker applications." 39.

Great care must be taken not to let even the most minute particle of veratria come in contact with the conjunctiva, since such an occurrence would produce great irritation.

*Case* 1. A lady, aged 55, had been afflicted with tic douloureux in the cheek, forehead, and eye-brow, for 36 years. The paroxysms generally came on once a week, and the intervals were never longer than 14 days. Her sufferings in the attacks were extreme; but in the intervals she was perfectly well. She was ordered to keep the bowels open by means of a pill, and at the commencement of each paroxysm, to take a small dose of acetate of morphium, to be repeated every half hour till the pain abated. She persevered in these means for two months, "and experienced considerable relief." But although the paroxysms were moderated, they were not subdued, and the intervals were not lengthened. She was then ordered to take small doses of the strychnine for a paralytic affection of the eye-lid, and continued it till convulsive twitches took place, yet without any beneficial effect. The veratria ointment (20 grains to the ounce) was then rubbed on the forehead and side of the face every day till the pain ceased. This was effected in fifteen minutes. The paroxysm returned in two hours, and the frictions were renewed, with extinction of the pain. To this there succeeded an interval of ten days' complete ease, when the attack returned as violent as before. The application was marked still more by relief than formerly. After this the patient had only one or two slight accessions of the tic. It is now four months since they ceased. The paralysis of the eyelid disappeared during the remedial process.

*Case* 2. A gentleman, aged 40, had laboured under tic douloureux in the right side of the face and forehead, for 16 years. There were few intermissions. His digestive organs were somewhat deranged, and he was ordered to take small doses of blue-pill with Epsom salts. The veratria ointment was also rubbed twice a day over the seat of the pain. In the course of four or five days he felt much better. He was directed to apply the ointment only when threatened with a paroxysm. At the end of four weeks he was cured.

*Case* 3. A lady, 48 years of age, had severe tic douloureux for 22 years in the face. She was treated in the same way as the preceding patient. After the third friction the pain ceased, and never afterwards returned.

*Case* 4. A lady, 35 years old, had suffered severely, and almost without intermission, for eighteen months, with tic in the cheek and forehead. As her general health seemed good, no medicine was prescribed, except the veratria ointment. The first friction cured her.

*Case* 5. A lady, 25 years of age, had tic for seven years, in the site of the supra-orbitar foramen. The paroxysms varied in length, from 16 hours to two days, the intervals being from ten days to three weeks. All means had been tried, without any permanent benefit. The digestive functions were disordered, and laxatives with blue pill were prescribed for a week. The carbonate of iron was tried for six weeks, without any benefit. Then the veratria ointment was rubbed on the eyebrow and forehead. The cure appears to have been complete.

*Case* 6. A lady, æt. 26, hysterical, had tic douloureux in the left eyebrow, since her fifteenth year. The paroxysms are usually once a month, but occasionally take place oftener, from alternations of temperature. She rubbed on the ointment, of usual strength, till the pain ceased, which it did in half an hour. Six months have now elapsed without any return of the paroxysm.

Several other cases of neuralgia of the face, head, back, &c. all apparently cured by this magic ointment.

### Rheumatism.

If veratria be capable of curing tic douloureux, it may, *a priori*, be conjectured, that it will be equally efficacious in chronic rheumatism—a disease so near akin to neuralgia. Dr. Turnbull observes that, in the acute form of the disease he has only tried it in two or three cases; but in these it was as beneficial as in the chronic. The ointment was rubbed directly on the inflamed surface, " and instead of producing any additional irritation, the inflammation and swelling rapidly subsided, and the pain was quickly subdued." Truly we have now nearly all we want in medicine!

" In lumbago, sciatica, rheumatic affections of the muscles over the chest, or in other parts, a removal of the symptoms is effected almost immediately by the first friction; and in more obstinate cases, a few more will, in general, have the desired effect." 61.

The cases we may pass over. Nine are detailed.

### Paralysis.

Those partial or local paralyses observed in the eyelids or face, where tic douloureux had existed, gave way under the use of veratria for the latter complaint. This led our author to an extension of the remedy to other cases of paralysis—and the results have been " so far favourable."

" In the cases referred to, the disease existed in different degrees of severity; in two or three, the patients had almost entirely lost the power of motion in one side of the body, but recovered it again, by making use of frictions with the ointment over the affected extremities, and more particularly along the course of the nerves. The greater number has, however, consisted of slight paralytic affections, situated in certain muscles, existing either by themselves or in conjunction with other affections, and especially with tic douloureux.

From the more extensive nature of the disease in the former cases, these have required the treatment to be continued for some little time; but in the latter, one or two applications have been successful in restoring the muscles to their healthy action.

In either instance, after the frictions have been made, there is a degree of warmth and tingling felt in the parts; gentle indeed at first, but becoming more and more manifest at each successive application, until the nerves of the affected parts are stimulated to a degree sufficient to enable them to resume their functions.

The proportions of the veratria employed ought, in most cases, to vary with the severity of the affections to be treated by it, and also, according to the extent of surface over which it has to be rubbed; but, as a general direction, fifteen or twenty grains to an ounce of lard will be sufficiently strong, and frictions of ten to twenty minutes duration will answer every purpose." 74.

Want of space compels us to pass over the cases, four in number. Some of them are far from convincing.

The fifth chapter is on the external use of veratria in dropsy. The forms of the disease were those of "hydrothorax, ascites, anasarca, and dropsy of the ovaries." "In all these it has been found of service, but particularly in the first three, several cases of which have been cured by it in a week or two, even when the severity of the symptoms was such as to threaten the life of the patient within a few hours." We cannot doubt the facts or even the assertions of an author; but certainly we would doubt the evidence of our own senses, if such cures of hydrothorax and ascites were to be performed before our eyes. The facts cannot, however, remain long in doubt, since the hopes generated by our author's statements, will stimulate thousands to test the miraculous remedy in every way. If we are not authorized to doubt, we must, at least, claim the privilege to fear!

Dr. T. acknowledges that encysted dropsies have resisted the veratria more obstinately than the other kinds; yet a few cases have been cured. The following passage is rather a qualifier.

"There appears to be two states of the disease, in both of which the veratria is of use, though in different degrees, when applied to the surface of the skin. The one, where the pathological condition of the organs upon which it has depended has been removed; and where, nevertheless, the aqueous effusion, from want of action in the absorbing vessels, remains: the other, where the organic change is such as will admit of no remedy. In the former instance, the veratria has succeeded in restoring the patient in a short time to a state of health; but in the latter, such an effect is not, of course, to be expected; yet, even where this is the case, considerable relief may be obtained from its employment, after other means have failed.

It is, therefore, indispensably necessary, before the veratria be applied, that every attention be paid to the state of all the organs, upon a derangement of which, either in structure or function, the effusion may depend; otherwise, the anticipated effects will not be produced. If, after a careful examination, nothing wrong, of importance, can be detected, the ointment may then be had recourse to, and probably with success; but if the contrary be the case, the diseased state, whatever that may be, should, if possible, be first removed, and then the treatment may be proceeded with. In some instances particularly of encysted dropsy, the presence of an unhealthy condition of the system totally unconnected with the disease itself, has very much impeded the action of the medicine, and has required removal, before it could go on without interruption." 81.

When dropsy depends, as it always does, on derangement in structure or function, the removal of the cause will generally prove the removal of the effect, even without veratria; and as Dr. T. very wisely directs us to "remove the diseased state, whatever that may be," before we apply the veratria, we may very readily believe that the application of the specific has been often followed by the subsidence of dropsy. It is not impossible that error has crept in through this channel, occasionally, in Dr. Turnbull's practice, without his suspicions being awakened to the source of it.

"As the frictions should, if possible, be made over the whole surface under which the effusion exists, and as this must vary with the situation and extent which it occupies, no prescription applicable in every instance can be given, except that the quantity of the ointment rubbed in each time, should not, in adults, contain less than two, nor more than four or five grains of veratria; and the friction should be continued for about twenty minutes, and repeated once or twice a-day, according to the impression produced; this in general begins to shew itself in a few hours; but not in the manner in which our knowledge of the consequences following from the medicine when given internally would lead us to anticipate." 82.

This veratria is, indeed, an extraordinary substance. Given internally, it vomits and purges—but, rubbed on the skin, it proves diuretic, and constipates the bowels.

"The quantity of urine evacuated by the patient, in some instances, almost exceeds belief, and might be considered as accidental, were it not that it has been observed to be an invariable occurrence. This circumstance is followed by an almost immediate subsidence of the swelling, which goes on gradually to diminish until none of it is left; the patient then gains strength daily, and soon acquires his former degree of health." 82.

We shall abridge a case, by way of illustration.

*Case.* J. B. Esq. of Pocklington near York, aged 30, came under Dr. T.'s care in the Summer of 1830, having then laboured under ascites for a year, without any advantage from medicine. He was now anasarcous throughout, with large effusion in the abdomen. The thoracic organs were very much impeded in function, with cough, dyspnœa, irregular pulse, and sense of suffocation. The urinary secretion was under a pint in the twenty-four hours. Our author pursued a plan previously acted on, viz. a course of mercury, squill, digitalis, colchicum, and various diuretics for six weeks, without any benefit.

"In this emergency it was resolved upon to make trial of the veratria externally, and a box of ointment made with four grains of the alcaloid and an ounce of lard, was accordingly directed to be rubbed over the surface of the abdomen at bed-time. The whole quantity was applied; and in the course of the night, and following morning, the patient evacuated no less than eight pints of urine, which had caused a marked diminution of the swelling, both in the abdomen and extremities, and was attended with considerable relief to the breathing and circulation; but, along with these effects, the medicine had caused such an alarming prostration of strength as to render the administration of stimulants absolutely necessary, for three days before the ointment could be repeated : at the end of that time, when the patient appeared somewhat recovered from his weakness, a fresh quantity was prescribed, in which, however, a less proportion of the veratria was used, owing to the violent constitutional symptoms caused by the first. On this occasion, 2 grains only were rubbed on, yet the diuretic effects were scarcely less marked than before ; and these were again accompanied by a degree of debility which, although not so great as in the preceding instance, still made it a matter of necessity to repeat the stimulants, and to delay the third application for five or six days.

On both occasions, after the first effects of the ointment had subsided, the quantity of urine diminished considerably, but the swelling became daily less in magnitude, and the patient went on improving in a manner that could not have been anticipated. On the fifth or sixth day from the second rubbing, a third, with an ounce of ointment, containing two grains of veratria, was directed to be made use of, as before; and from this time, the dropsy rapidly disappeared: the patient gained strength sufficient to enable him to take active exercise ; and at the end of three weeks from the first application of the veratria, he was completely cured, and has since had no return of the disease." 87.

There is a short and concluding chapter on veratria in gout, amaurosis, &c. on which we need not dwell. Our author's experience in these diseases has not been sufficiently extensive to warrant an arrangement under distinct heads; but he has seen enough to prognosticate that, in gout especially, veratria promises to be of great service.

"The best time to employ it appears to be at the very commencement of the accession ; and at this period, friction for 20 minutes made over the seat of the pain, with an ointment consisting of twenty grains of veratria to an ounce of lard, has cut short the paroxysm, and has at once enabled the patient to make use of the affected joints." 93.

We have now given a very extensive and fair analysis of the work, and sincerely hope that the experience of others may confirm that of our author. We have commenced a trial of the remedy ourselves, but cannot give any results in the present review. We hope soon to be enabled to report upon the remedy. Meantime, it is necessary to guard against adulterated veratria. The extreme dearness of the article will lead to sophistication, and, therefore, it should only be procured from houses of the very first respectability, regardless of the price. It is probable that the salts of veratria will come into use instead of the base. The only one that has yet been tried is the sulphate, which appears to be far more energetic than the veratria itself—for even half a grain, when rubbed on the skin, produces a degree of heat and tingling amounting to pain.

